# Comparison of Tensile and Compressive Properties of Carbon/Glass Interlayer and Intralayer Hybrid Composites

**DOI:** 10.3390/ma11071105

**Published:** 2018-06-28

**Authors:** Weili Wu, Qingtao Wang, Wei Li

**Affiliations:** 1 College of Textiles, Donghua University, No. 2999, Northern Renmin Rd., Songjiang District, Shanghai 201620, China; 1152003@mail.dhu.edu.cn (W.W.); a19870628wqt@126.com (Q.W.); 2 Key Lab of Textile Science & Technology, Ministry of Education, Shanghai 201620, China; 3 Center for Civil Aviation Composites, Shanghai 201620, China

**Keywords:** carbon/glass hybrid composites, interlayer hybrid, intralayer hybrid, tensile properties, compressive properties, ratio of tensile/compression strength

## Abstract

Tensile and compressive properties of interlayer and intralayer hybrid composites were investigated in this paper. The tensile modulus and compression modulus of interlayer and intralayer hybrid composites are the same under the same mixed ratio, the tensile strength is much superior to the compression strength, and while the tensile modulus and strength increase along with the carbon fiber content, the compression values change slightly. The influence of stacking structures on the tensile and compressive strengths is opposite to the ratio of T/C (tensile/compression) strength for interlayer hybrid composites, and while the tensile and compression strengths with glass fiber sandwiching carbon fiber can reach the maximum value, the ratio of T/C strength is minimum. For structures with carbon fiber sandwiching glass fiber, or with asymmetric structures, the tensile and compressive strengths are at a low value. For intralayer hybrid structures, while the carbon/glass (C/G) dispersion degree is high, the tensile and compression strengths are low. The experimental tensile and compressive strengths for interlayer and intralayer hybrid composites are greater than the theoretical values, which demonstrates that strength conforms well to the positive hybrid effect. The tensile fracture strain is greater than the compression fracture strain for hybrid composites, with both of them basically maintained at the same level.

## 1. Introduction

Due to high price of carbon fiber, hybrid composites composed of two or more fiber materials have been developed for cost reduction [1]. Hybrid composites not only embody the advantages of a single fiber, but can achieve complementary advantages of two materials; excellent physical and mechanical properties make hybrid composites widely applicable in many fields [2,3,4,5,6,7]. Currently, extensive work centers on the mechanical properties of hybrid composites [8,9,10,11,12,13,14]. Manders et al. [13] investigated tensile properties of carbon/glass hybrid composites with various fiber contents and layer structures to explore the impact factors of the hybrid effect. Phillips L N [15] studied hybrid composites and found that its mechanical properties, including tensile, impact, and fatigue properties, are better than in pure carbon fiber or glass fiber composites. Dong et al. [16,17] revealed that the hybrid effect is obvious since there is a distinct Young modulus difference between carbon and glass fiber, and an optimal mixed ratio has been obtained via theoretical analysis. Li et al. [18] studied the compression properties of UHMPEF/carbon hybrid composites and found the experimental compressive strength is basically consistent with theoretical values via the rule of mixture (ROM), and compressive fracture strain exhibits the positive hybrid effect.

In addition, a difference between the tensile and compressive properties of fiber and composites exists due to fiber buckling and production problems, which have been confirmed in many papers, with some authors having studied the tensile and compressive properties from the fiber viewpoint. Greenwood et al. [19] studied aramid fiber and found a highly oriented crystalline cellulose structure makes the tensile strength of fiber stiff, which makes it easy to form a kink band under the compressive loading contributing to compressive strength only accounting for 20% of the tensile strength. Oya et al. [20] found the compressive strength of PAN (polyacrylonitrile)-based carbon fiber only accounts for 30–50% of the tensile strength, and the compression modulus in the longitudinal direction was calculated by the Euler buckling method and accounts for 50% of the tensile modulus. Bos et al. [21] found that flax fiber has a high compression-to-tensile ratio of 80% in the loop test. Meng et al. [22] found the average ratio of tensile to compressive modulus is 0.9 due to misalignment of fiber and production problems (porosity content). In addition, the inclination angle of fiber was measured using an indirect method and the inclination angle distribution of fiber was given.

In addition to the fiber investigation, some researchers studied the tensile and compressive properties of composites. Zobeiry et al. [23] developed a model to explain the failure mechanism of composite under tension and compression, where the tensile failure is mainly due to the kink band, which is the reason for the formation of fiber failure and resin fracture or yielding. The ratio of tensile and compressive strength of flax and bamboo composites was found to be 60%, and the compression properties of coir fiber composites were even better than the tensile properties [24]. Tensile and compressive testing combined with computed tomography (CT) were adopted to investigate the tensile failure in a laminate and the fiber wrinkling under compression [25]. Mujika et al. [26] determined the ratio of tension and compression moduli by two experiments of four-point bending. Liu et al. [27] studied the tensile/compressive properties of PVA (polyvinyl alcohol)-based, cement-matrix composites. The ratio of tensile/compressive strength was regarded as an important parameter to evaluate the brittleness of cement; a smaller ratio of value indicates a larger brittleness and a smaller toughness. Choi et al. [28] studied the tensile and compression properties of several concrete composites and found the ratio of tensile to compressive strength is 19.8% on average, which is almost twice that of normal concrete. Hartl et al. [29] confirmed that the compression/tensility of short glass fiber-reinforced polypropylenes has a stress-strain asymmetric behavior, and the ratio of compressive to tensile strength can reach 1.3 due to the fiber misalignment. The tensile and compressive properties of 3D woven carbon-fiber-reinforced composites were studied; while the tensile modulus and compression modulus were nearly the same, the tensile strength was far greater than the compressive strength, which is mainly due to the obvious crimp of 3D fabric. The resin breaks easily while subjected to the compressive loading, then delaminates, and then the sample fails [30]. Prabhakaran et al. [31] compared the tensile and compressive properties of hybrid composites and found the tensile and compressive moduli and strengths are consistent with previous conclusions [30].

This paper systematically designed carbon/glass interlayer and intralayer hybrid structures and studied the effect of mixed ratios and hybrid structures on the tensile and compressive properties of hybrid composites. In addition, ratios of tensile/compressive (T/C) strength were studied and analyzed.

## 2. Materials and Methods

### 2.1. Experimental Materials

**Figure 1:**
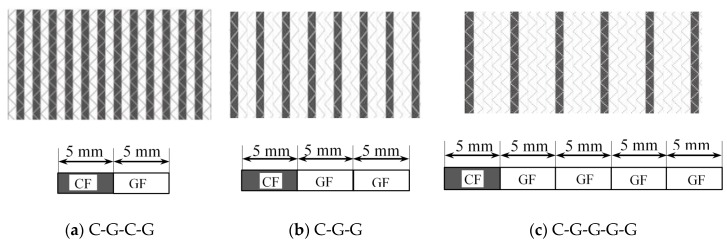
Schematic structures of three kinds of intralayer hybrid NCFs.

Carbon fiber, glass fiber, and epoxy resin were supplied by TORAY Inc. (Tokyo, Japan), CPIC Inc. (Chongqing, China), and SWANCOR Inc. (Shanghai, China), respectively. Five kinds of unidirectional Non-Crimp Fabrics (NCF), including pure carbon and glass fiber fabric and three kinds of hybrid fabrics, were designed and manufactured. Mechanical parameters of raw materials and fabric structures are given in Table 1 and Table 2. Structure diagrams of intralayer hybrid fabric are shown in Figure 1.

### 2.2. Layer Structures Schemes of Interlayer Hybrid Structures

With regard to interlayer hybrid composites, four C/G hybrid ratios with various hybrid structures were designed by altering stacking sequences of pure carbon and glass fiber fabric. Interlayer hybrid structures are shown in Table 3. For interlayer hybrid structures with various C/G hybrid ratios, the number of layers in a laminate was not the same. When C:G = 1:2, a laminate contained only three layers; for C:G = 1:4, a laminate contained five layers. 

### 2.3. Schemes Design of Intralayer Hybrid Structures

Three kinds of intralayer hybrid fabrics were used, and dispersion degrees were achieved through variations in dislocation arrangements of carbon and glass fiber bundles in various layers as shown in Table 4. The number indicates the dispersion degree; for example, for the structure [C-C-G-G]-0, the carbon and glass bundles were aligned with the upper and lower layers, and the structure [C-C-G-G]-0.5 indicates the fabric translated horizontally the half-width of one bundle.

### 2.4. Experiments

Vacuum-assisted resin transfer molding process (VARTM) was used to prepare composite laminates; the detailed process is referred to in reference [32]. Tensile testing and compression testing were conducted according to the ASTM D3039 Standard [33] and ASTM D6641 [34] separately. Due to variations in failure speeds and modes of carbon and glass fiber, the force attenuation rate was set to 50% as the experimental end parameter.

**Figure 2:**
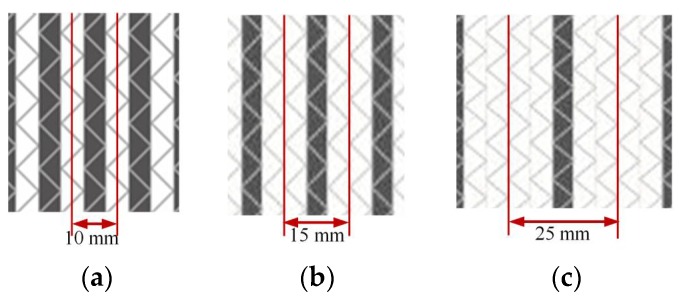
Schematics of cutting location for intralayer hybrid composites: (**a**) [C-C-G-G]; (**b**) [C-G-G]; and (**c**) [C-G-G-G-G].

Five specimens of each laminate were tested, and sample width of tensile and compression testing for interlayer hybrid composites based on the standards was set to 15 mm and 13 mm, respectively. The specimen width for intralayer hybrid composites was set to the width of a minimum repeating unit. In order to avoid the effect of boundary condition on the mechanical properties, carbon and glass bundles in a specimen were distributed symmetrically. Specimen width and cutting positions of three intralayer hybrid composites are shown in Figure 2.

The fiber volume content was kept at 50% and the thickness of each layer in a laminate was 0.8 mm, therefore, dimensions of each laminate were not same and depended on the number of layers and the hybrid forms. The size parameters of composites are listed in Table 5.

## 3. Results and Discussions

### 3.1. Tensile and Compressive Properties of Interlayer Hybrid Composites

**Figure 3:**
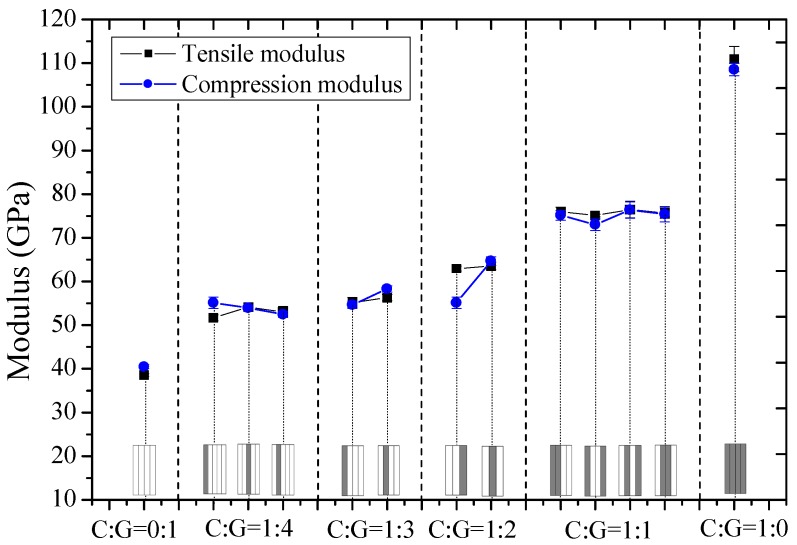
Tensile and compression moduli of interlayer hybrid composites with various stacking sequences and C/G hybrid ratios.

**Figure 4:**
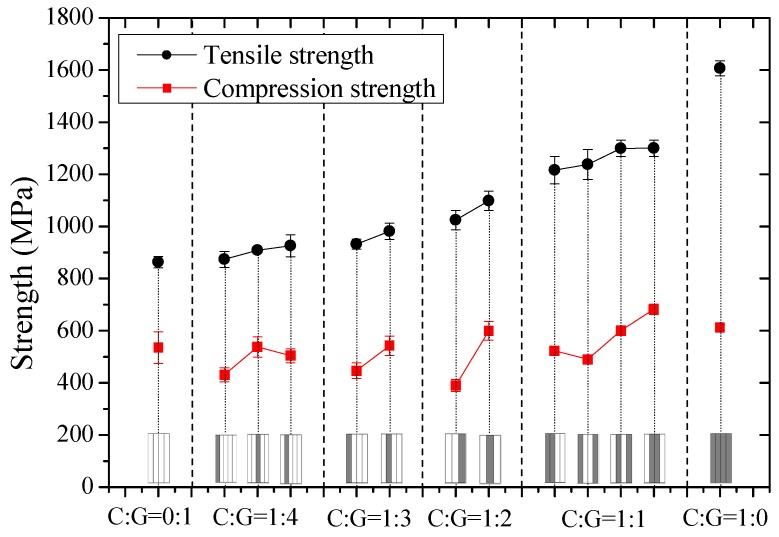
Tensile and compression strength of interlayer hybrid composites with various stacking sequences and C/G hybrid ratios.

**Figure 5:**
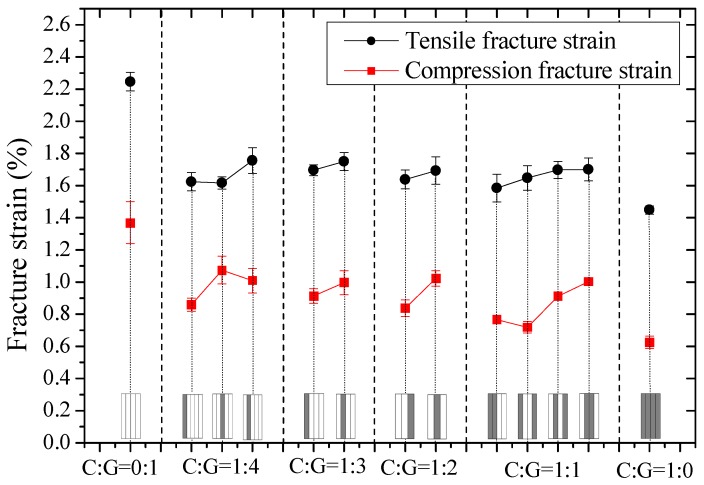
Tensile and compression fracture strain of interlayer hybrid composites with various stacking sequences and C/G hybrid ratios.

Tensile and compression moduli, strengths, and fracture strains of interlayer hybrid composites with various mixed ratios and stacking sequences were compared in Figure 3, Figure 4 and Figure 5, and the effect of hybrid ratios and layer structures on the tensile and compressive properties were analyzed.

As seen from Figure 3, the tensile and compression moduli of interlayer hybrid composites are very close and mainly determined by the factor C/G mixed ratio; the tensile and compression moduli increase gradually as the carbon fiber content increases. Under the same C/G mixed ratio, tensile and compression moduli of interlayer hybrid composites with various hybrid structures change slightly.

Comparison of tensile and compression strengths of interlayer hybrid composites is shown in Figure 4. It was found that the tensile strength is superior to the compression strength, moreover, the tensile strength increases obviously along with the carbon fiber content while the compression strength only changes slightly. Fiber-reinforced composites consist of fiber and resin matrix; while subjected to the tensile loading, fiber along the longitudinal direction mainly assumes the force, and the tensile strength is determined by the tensile properties of the reinforced fiber. However, when composites are subjected to a compression loading, the fiber and the resin assume the force, which will affect the compression strength of composites in common, therefore, the tensile and compression properties of composites exhibit a difference. 

Under the same C/G mixed ratio, the tensile and compression strengths of interlayer hybrid composites are highly correlated with the stacking sequences. Excellent strength can be achieved for glass fiber sandwiching carbon fiber, like [G/G/C/G/G], [G/C/G/G/G], [G/C/G], [G/C/C/G]. For symmetric structures with carbon fiber sandwiching glass fiber like [C/G/G/C], or asymmetric interlayer structures, like [C/G/G/G/G], [C/G/G/G], [C/G/G], [C/C/G/G], the tensile and compression strengths are maintained at a low level.

Tensile and compression fracture strains of interlayer hybrid composites are shown in Figure 5. The tensile fracture strain is greater than the compression fracture strain, and both the strains drop slightly as the carbon fiber content increases. 

### 3.2. Tensile and Compressive Properties of Intralayer Hybrid Composites

**Figure 6:**
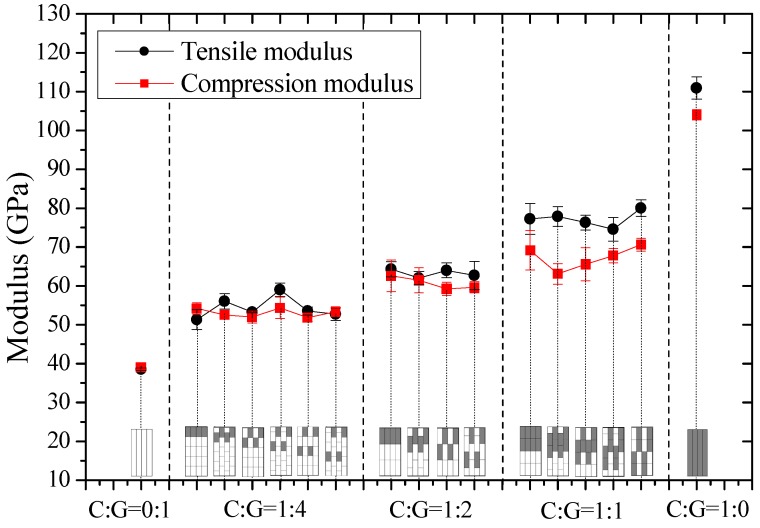
Tensile and compression moduli of intralayer hybrid composites with various stacking sequences and C/G hybrid ratios.

**Figure 7:**
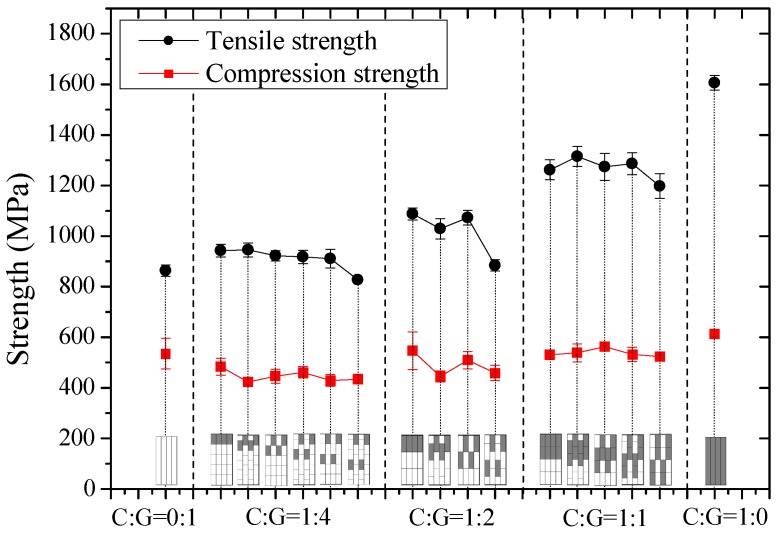
Tensile and compression strengths of intralayer hybrid composites with various stacking sequences and C/G hybrid ratios.

**Figure 8:**
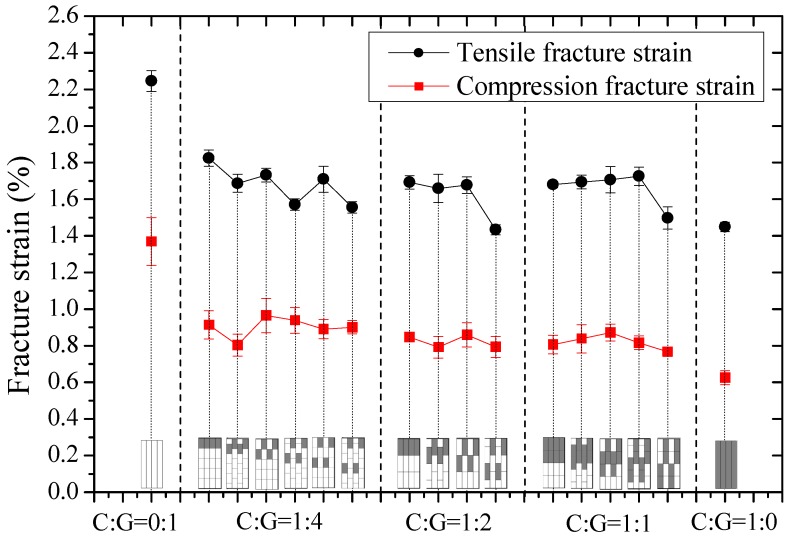
Tensile and compression fracture strains of intralayer hybrid composites with various stacking sequences and C/G hybrid ratios.

In this part, tensile and compression moduli, strengths, and fracture strains of intralayer hybrid composites with various mixed ratios and hybrid structures were investigated and results are shown in Figure 6, Figure 7 and Figure 8.

In Figure 6, it was concluded that the tensile and compressive moduli increase as the carbon fiber content increases for intralayer hybrid composites. Under the same mixed ratio, the modulus maintains at the same level which is independent of the hybrid structures. The tensile and compression moduli of various layer structures are basically identical with little carbon fiber, like C:G = 1:4, 1:2, however, the compression modulus of C:G = 1:1 tends to be slightly lower than the tensile modulus.

Figure 7 revealed that the tensile strength of intralayer hybrid composites is much greater than the compression strength. The difference is around 100%, and the tensile strength increases as the carbon fiber content increases, while the compression strength appears to be independent of the hybrid structures and the hybrid ratios. Under the same mixed ratio, the effect of hybrid structures on the tensile and compression strengths is small, and the compressive strength is at a lower level. With a high C/G mixed dispersion degree, such as [C-G-G-G-G]-2.5, [C-G-G]-1.5, and [C-C-G-G]-2, the tensile strength drops; it is assumed the carbon fiber breakage results in the glass fiber damage leading to a comparatively low strength of intralayer hybrid composites.

In Figure 8, it can be concluded that the tensile fracture strain of intralayer hybrid composites decreases slightly as the carbon fiber content increases, and the tensile strain is higher than the compressive strain. In addition, the fracture strain tends to be lower if the C/G dispersion degree is high.

### 3.3. Comparison of Tensile and Compressive Properties of Interlayer and Intralayer Hybrid Composites

Ratio of tensile/compression (T/C) strength is defined to illustrate the relationship between tensile and compressive properties of hybrid composites as formula (1). A small T/C ratio indicates the tensile strength is close to the compression strength.

(1) $$R_{T:C}=\frac{\sigma_{T}}{\sigma_{C}}$$

where *R_T:C_* denotes the ratio of T/C strength, *σ_T_* and *σ_C_* are the tensile strength and compression strength of composites (MPa), respectively.

**Figure 9:**
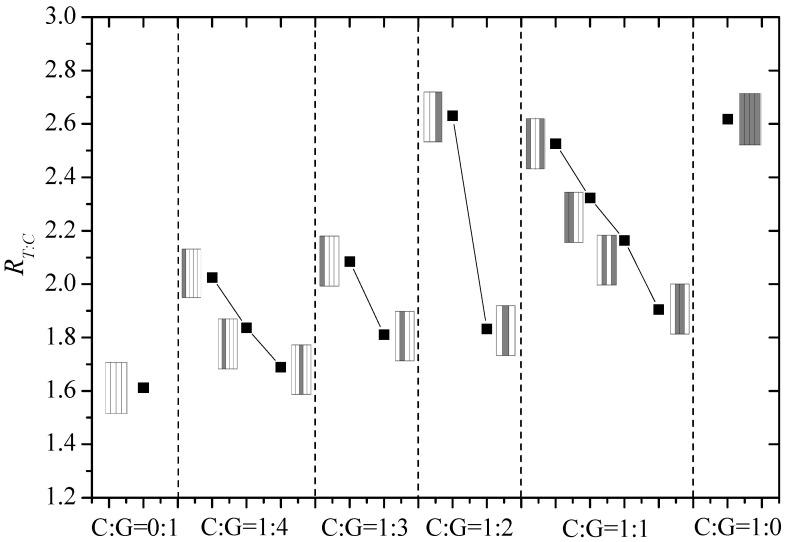
Ratio of T/C strength of interlayer hybrid composites.

Figure 9 reports ratios of T/C strength for interlayer hybrid composites with various mixed ratios and hybrid structures. It can be found that ratios of T/C strength increase along with the carbon fiber content overall, which indicates the difference between tensile and compression strengths becomes larger. Under the same C/G mixed ratio, ratios of T/C strength with glass fiber sandwiching carbon fiber, such as [G/G/C/G/G], [G/C/G/G], [G/C/G], [G/C/C/G], are the minimum. With regard to structures with carbon fiber sandwiching glass fiber, like [C/G/G/C], or with carbon fiber locating in one surface, like [C/G/G/G/G], [C/G/G/G], [G/G/C], ratios of T/C strength can reach up to a high level.

**Figure 10:**
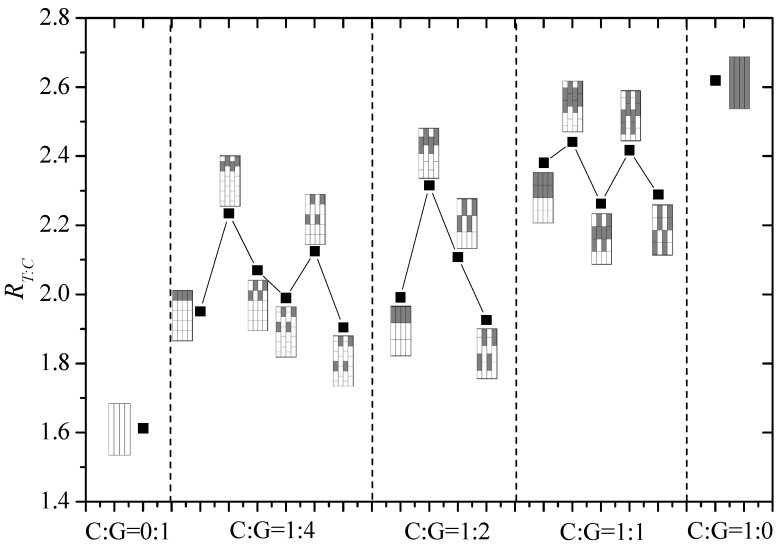
Ratio of T/C strength of intralayer hybrid composites.

Ratios of T/C strength of intralayer hybrid composites with various mixed ratios and hybrid structures are shown in Figure 10. There is not a clear law to describe the relationship between the ratio of T/C and the carbon fiber content, however, under the same C/G hybrid ratio, with a small dispersion degree like 0.5, structures have high ratios of T/C, while the composites with a high dispersion degree can reach low ratios of T/C.

In order to evaluate the hybrid effect efficiently, ROM (Rule of Mixture) is introduced. ROM is a method to calculate mechanical properties of hybrid composites according to the ratio of two materials:

(2) $$E_{ROM}=\frac{E_{C}V_{fC}}{V_{fC}+V_{fG}}+\frac{E_{G}V_{fG}}{V_{fC}+V_{fG}}$$

where $E_{ROM}$ is the compression modulus of hybrid composite (GPa); $E_{C}$, $E_{G}$ are the compression moduli of carbon fiber and glass fiber composite (GPa), respectively; $V_{fC}$, $V_{fG}$ are the volume contents of carbon fiber and glass fiber composite, respectively.

**Figure 11:**
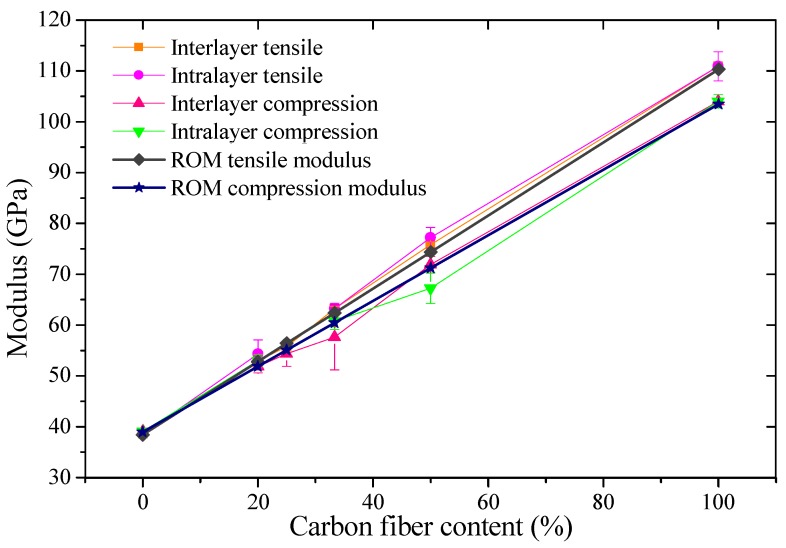
Experiment and theoretical values of tensile and compression moduli of hybrid composites.

Experimental and theoretical values of tensile and compression moduli for interlayer and intralayer hybrid composites are shown in Figure 11. It was found that tensile and compression moduli increase linearly along with the carbon fiber content, and the compression modulus is lower than the tensile modulus, but almost the same.

**Figure 12:**
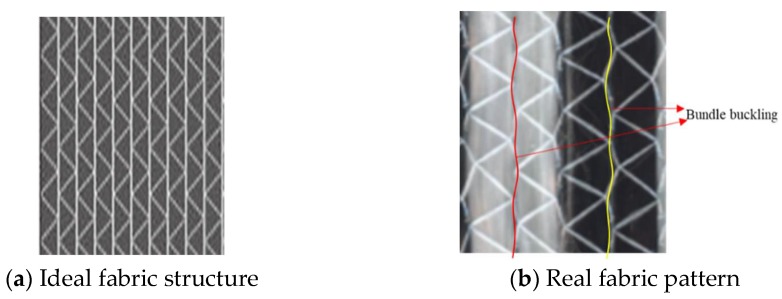
NCF Fabric.

The tensile modulus is superior to the compression modulus resulting from the structure of NCF fabric. Figure 12a shows the ideal structure of the NCF fabric and Figure 12b is the real fabric. Due to the weaving process, warp yarns in a fabric are constrained via stitching yarns in an unbalanced force, leading to a certain buckling of warp yarns. As the composite is subjected to a tensile loading, fiber and yarns along the longitudinal direction are gradually stretched, and high modulus of fiber will be fully utilized. However, as the specimen assumes a compressive force along the fiber axis, the buckling fiber and yarns have no chance to stretch, therefore, the compression modulus is low.

Theoretical stress and strain are not a simple superposition according to the ROM due to difference in fracture strain of carbon and glass fiber. As the compressive strain reaches the fracture strain of carbon fiber, carbon fiber fails first, and glass fiber assumes the residual load until the specimen fails. Calculations of compression stress are shown in (3) and (4).

(3) $$\mathrm{Before}\;\mathrm{carbon}\;\mathrm{fiber}\;\mathrm{fracture}:\;\sigma_{\mathrm{HY}}=\left(V_{C}E_{C}+V_{G}E_{G}\right)\varepsilon$$

(4) $$\mathrm{After}\;\mathrm{carbon}\;\mathrm{fiber}\;\mathrm{fracture}:\;\sigma_{\mathrm{HY}}=V_{G}E_{G}\varepsilon$$

where $\sigma_{\mathrm{HY}}$ is the compression stress of hybrid composite (MPa); $V_{C}$, $V_{G}$ are the volume contents of carbon fiber and glass fiber composite, respectively; $E_{C}$, $E_{G}$ are the compression moduli of carbon fiber and glass fiber composite (GPa), respectively; and ε is the strain of hybrid composite.

**Figure 13:**
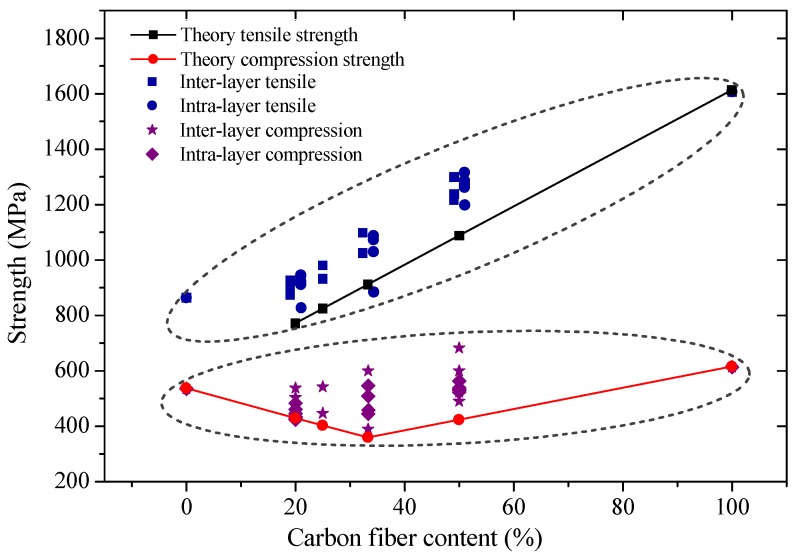
Experimental and theoretical values of tensile and compression strengths of hybrid composites.

The relationship between the carbon fiber fraction and strength is illustrated in Figure 13. Comparisons of experimental and theoretical results showed that tensile strength of hybrid composites appears to increase much more obviously than the compressive strength along with the carbon fiber content, and the compressive strength tends to drop first and then rise later. Experimental values are higher than theoretical ones, which indicates the tensile and compression strengths of hybrid composites conform well to the positive hybrid effect.

**Figure 14:**
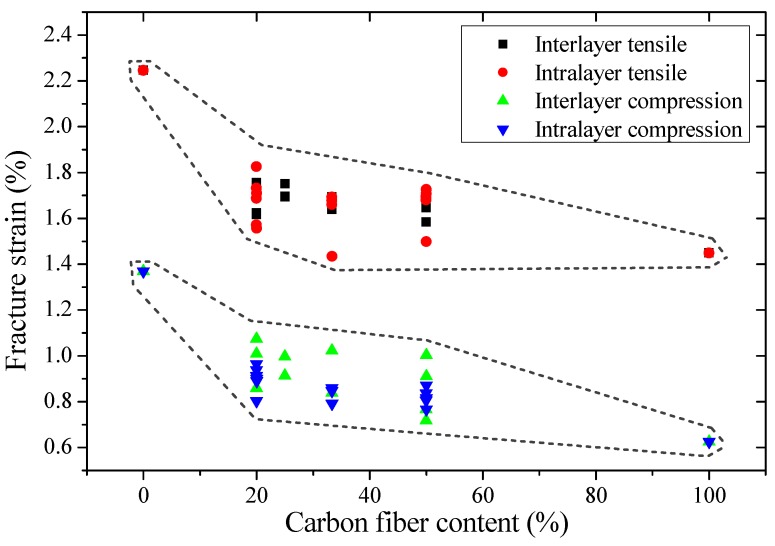
Tensile and compression strengths of interlayer and intralayer hybrid composites obtained by experiment and ROM.

When the carbon fiber content ranges from 0% to 20%, the tensile and compression fracture strains of hybrid composites, shown in Figure 14, decrease slightly. Nevertheless, the decrease of fracture strain drops slowly with the carbon fiber content ranging from 20% to 100%. As the fracture energy release of little carbon fiber is not sufficient to cause a complete failure of the specimen rapidly, the damage exhibits a progressive failure. When the carbon fiber content increases to a certain fraction, the fracture energy release of carbon fiber leads to a rapid failure of hybrid composites, and the fracture strain is close to the pure carbon fiber composites.

## 4. Conclusions

Tensile and compressive properties of interlayer and intralayer hybrid composites were studied in this work. 

(1) The tensile modulus and compression modulus of interlayer and intralayer hybrid composites are basically the same under the same mixed ratio, and they increase linearly along with the carbon fiber content. Variations in hybrid structures have no effect on the tensile and compression moduli. 

(2) Analysis of tensile strength and compression strength of interlayer and intralayer hybrid composites revealed that the tensile strength is significantly greater than the compression strength resulting from the warp bulking. The tensile strength increases along with the carbon fiber content, and the compression strength changes slightly. 

(3) The tensile fracture strain is greater than the compression fracture strain for interlayer and intralayer hybrid composites; both of them drop slightly as the carbon fiber content increases, and for intralayer hybrid composites, the strain tends to be lower as the C/G dispersion degree is high.

(4) Ratios of T/C strength for interlayer and intralayer hybrid composites increase as the carbon fiber content increases. For interlayer hybrid composites with the same C/G mixed ratio, the ratio of T/C strength with glass fiber sandwiching carbon fiber is the minimum. By contrast, the ratio of T/C strength with carbon fiber sandwiching glass fiber or with carbon fiber distributed in one side can reach the maximum level. For intralayer hybrid composites, the effect of stacking structures is small, and for some dispersion degree, the structures can achieve a low ratio of T/C. Tensile and compression strengths of hybrid composites are larger than the theoretical values, which conform to the positive hybrid effect.

## Figures and Tables

**Table 1:** Constituent materials and selected properties.

Material	Tensile Strength (MPa)	Tensile Modulus (GPa)
CPIC ECT469L-2400 glass fiber	2366	78.7
TORAY T620SC-24K-50C carbon fiber	4175	234
SWANCOR 2511-1A/BS epoxy resin	73.5	3.1

**Table 2:** Specifications for hybrid fabric.

Fabric Type	Areal Density (g/m^2^)		Ratio of C/G
	Carbon Fiber	Glass Fiber	
carbon	728.3	0	1:0
glass	0	944.9	0:1
C-G-C-G	364.2	472.4	1:1
C-G-G	242.8	629.9	1:2
C-G-G-G-G	145.7	755.9	1:4

**Table 3:** Stacking configurations of interlayer hybrid structures.

Hybrid Ratio	Stacking Sequences			
C:G = 1:1	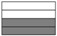	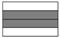	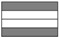	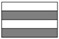
	[G/G/C/C]	[G/C/C/G]	[C/G/G/C]	[G/C/G/C]
C:G = 1:2	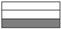	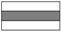		
	[G/G/C]	[G/C/G]		
C:G = 1:3	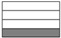	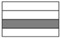		
	[G/G/G/C]	[G/G/C/G]		
C:G = 1:4	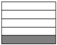	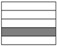	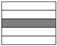	
	[G/G/G/G/C]	[G/G/G/C/G]	[G/G/C/G/G]	

**Table 4:** Stacking configurations of intralayer hybrid structures.

Hybrid Fabric	Stacking Sequences			
C-C-G-G	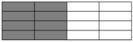	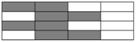		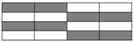
	[C-C-G-G]-0	[C-C-G-G]-1		[C-C-G-G]-2
	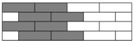	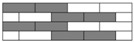		
	[C-C-G-G]-0.5	[C-C-G-G]-1.5		
C-G-G	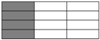	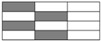	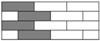	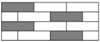
	[C-G-G]-0	[C-G-G]-1	[C-G-G-0].5	[C-G-G]-1.5
C-G-G-G-G	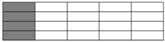	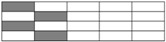		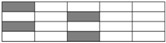
	[C-G-G-G-G]-0	[C-G-G-G-G]-1		[C-G-G-G-G]-2
	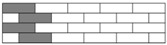	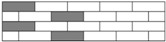		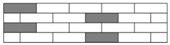
	[C-G-G-G-G]-0.5	[C-G-G-G-G]-1.5		[C-G-G-G-G]-2.5

**Table 5:** Size parameters of composites.

Laminate Structures	C/G Hybrid Ratios	Layers	Laminate Thickness/mm	Width/mm	Span/mm
pure carbon fabric	1:0	4	3.2	13	64
pure glass fabric	0:1	4	3.2	13	64
interlayer laminate	1:1	4	3.2	13	64
	1:2	3	2.4	13	48
	1:3	4	3.2	13	64
	1:4	5	4	13	80
intralayer laminate	1:1	4	3.2	20	64
	1:2	4	3.2	15	64
	1:4	4	3.2	25	64
